# Essential medicines concept and health technology assessment approaches to prioritising medicines: selection versus incorporation

**DOI:** 10.1186/s40545-023-00595-4

**Published:** 2023-07-13

**Authors:** Petra Brhlikova, Thilagawathi Abi Deivanayagam, Zaheer-Ud-Din Babar, Claudia Garcia Serpa Osorio-de-Castro, Rosângela Caetano, Allyson M. Pollock

**Affiliations:** 1 Population Health Sciences Institute, Newcastle University, Baddiley-Clark Bldg, Newcastle Upon Tyne, NE2 4AX UK; 2 Lancaster Medical School, Faculty of Health and Medicine, Lancaster University, Lancaster, UK; 3 Institute for Global Health, University College London, London, UK; 4 Department of Pharmacy, University of Huddersfield, Huddersfield, UK; 5 Departamento de Política de Medicamentos E Assistência Farmacêutica, Escola Nacional de Saúde Pública Sergio Arouca, Fundação Oswaldo Cruz, Rio de Janeiro, Brazil; 6 Departamento de Política, Planejamento E Administração Em Saúde, Instituto de Medicina Social, Universidade Do Estado Do Rio de Janeiro, Rio de Janeiro, Brazil

Growing expenditure on medicines is impacting the sustainability of health systems [1]. The global pharmaceutical market is estimated to grow at a rate of 3–6% annually through 2027, surpassing US$1.9 trillion by 2023. An average of 65 new drugs are expected to be launched per year, primarily oncology, immunological, anti-diabetic, and obesity drugs, resulting from a continuous stream of innovative products [2]. Medicines are also the biggest driver of out-of-pocket payments (OOPs) and catastrophic health expenditure globally, with spending on medicines creating a greater financial burden for households than spending on inpatient or outpatient services [3]. On the one hand, overdiagnosis, inappropriate prescribing and medicine use may lead to over-treatment, inappropriate treatment, and health hazards [4]. On the other hand, lack of access to affordable medicines is a significant barrier to accessing health care [5].

In this commentary, we look at two paradigmatic approaches to prioritising medicines for use in health systems—the Essential Medicines concept, upon which the process of selecting essential medicines is based, and Health Technology Assessment, which is the process for comparing individual medicines using an aggregate of analytical tools, mainly involving cost-effectiveness. The paper intends to highlight gaps in research and lack of evidence for these approaches mainly on their effectiveness in containing costs, ensuring access and appropriate medicine use.

## Medicines selection based on the WHO essential medicines concept

For the last four decades, the WHO has made strenuous efforts to enable universal access to affordable and essential medicines. In 1975, in response to growing concerns over the increasing number of medicines in the market and the need to ensure that government procurement and prescribers focus on key medicines to meet public health needs, the WHO took the discussion to the World Health Assembly [6]. It resulted in the development of the essential medicines (EM) concept, and its main strategy, the Essential Medicine List (EML), also known as the Model list. The EML is a limited list of medicines covering all therapeutic classes and is the most widely used tool for prioritising medicines.

The EM concept is a programme which covers all aspects of drug management, including procurement, storage, distribution, prescribing and use. It recognises the need to rationalise and prioritise the selection of effective medicines for proven health needs. The EM concept is rooted in evidence-based and priority need-based selection. Essential medicines are selected on the basis of population need, efficacy, safety, effectiveness, quality, and affordability and are deleted/deselected if better alternatives become available or if shown to be ineffective or harmful [7, 8]. The WHO also requires evidence of market registration, i.e. producers have a licence for manufacturing the drug for use.

Pricing of medicines and affordability to the health system and public purse underpins essential medicines policy [5]. The alignment between EML which guides medicine procurement, and thus availability, and treatment guidelines which support prescribing is crucial.

The concept has been voluntarily adopted and implemented by 156 out of 193 WHO member states [7], which develop and implement their own lists, inspired by the WHO EML. National EMLs may be further adapted for use at state, regional and district levels and within the different levels of the health system [7]. Tools for implementation of the list are Standard Treatment Guidelines (STGs), which may be developed prior to or after inclusion of medicines on to the list [9] and drug formularies, which include indications and information for the prescriber [10].

The budgetary impact on health systems in countries which adopt the EM concept is unknown. In many countries there is under-registration of essential medicines and many low-income countries do not procure all medicines on their EMLs. They may also use additional levels of prioritisation (Vital Essential Necessary, VEN classification) when budgets are tight. This economic classification is for example used at different levels of the health system in Jamaica, Trinidad and Tobago, and Uganda [11–13]. While in 1977, the EML selection criteria considered only off-patent, generic medicines, and emphasised the need to select effective and safe low-priced medicines [14], novel on-patent and high-cost medicines have been incorporated into the WHO EML in recent years. This further strain on the budgets of low- and middle-income countries and inclusion of medicines not yet approved by any stringent regulatory authorities have led some to question whether we are moving to a new definition of essential medicines [14].

## Medicines incorporation as a product of health technology assessment (HTA)

Some countries including the UK and US use Health Technology Assessment (HTA) as an alternative to assist public payers and health systems in decision-making. Developed in 1972 in the USA by the Office of Technology Assessment to provide health policymakers with a tool for comparative decision-making on policy alternatives, HTA focused on assessing the safety, efficacy, and cost-effectiveness of new imaging technologies, e.g. computerised tomography (CT). It was then applied to medical devices and drugs in the early 1980s, with the creation of the National Centre for Health Care Technology Assessment to advise the Medicare insurance programme on which technologies to include at the national level [15].

HTA may be used in place of or in addition to the EM concept in the process of selecting medicines for a list or incorporating medicines into the health system [14]. In developed countries the primary focus has been on novel medicines given their high cost. Pharmaceutical companies refer to HTA as the ‘fourth hurdle’ following the regulatory requirements of safety, efficacy, and quality [16, 17]. In this context, the starting point for HTA is the existence of evidence on an individual medicine, preferably quality evidence of safety, effectiveness and cost-effectiveness, but sometimes even low or moderate quality evidence, in contrast to the focus on population priority needs which is embedded in the EM concept. The assessment, usually through a cost-effectiveness or cost–utility analysis, results in either refusal to fund or incorporation of the medicine into the health system. HTA appraisals and tools vary in their definitions, criteria, and methods from country to country [18].

In the UK, ‘willingness to pay’ and not affordability to the system is the basis of incorporation. The HTA agency, National Institute of Care and Clinical Excellence (NICE), uses the incremental cost-effectiveness ratio (ICER) to calculate costs of the new medicine in relation to its expected health benefits [19]. Although budget impact on the whole system is calculated in parallel, it is not part of NICE decision-making and does not affect whether a technology is recommended for use in the public health system. Therefore, if a medicine provides significant health benefits at an acceptable cost for the individual patient treated, it will be recommended by the HTA agency, regardless of cost of implementation [19]. An approval from NICE does not mean the NHS is legally required to provide the recommended drug to the population; rather, resource impact considerations are left to local health providers.

HTA is a highly technical and costly process and usually involves econometric modelling which most countries do not have the capacity and financial resources to undertake [20–22].

## Tools for implementation

### Drug formularies and national reimbursement lists (NRL)

Formularies are defined by the WHO as “lists of pharmaceuticals permissible to use in a health insurance programme” [23]. The WHO first published a Model Formulary in 2002, to guide the effective use of the WHO EML [9]. A WHO Model Formulary was last published in 2008, after which only the EML exists as an international list [9].

Formularies are intended to reduce budget impact, by creating a select list of medicines with indications for use, and increase quality of care by reducing inappropriate prescribing of medicines [5]. Drugs are usually listed by therapeutic class, and details are given on use, dosage, adverse effects, contraindications, and warnings. Decisions on drugs to be included may be based on similar criteria to those used by National EMLs and HTA, i.e. safety, efficacy, and cost-effectiveness [24]. However, formularies vary in their purpose, composition, and use. Some countries develop formularies as by-products of their EMLs, while others are freestanding. The British National Formulary (BNF) lists all registered medicines. In other countries the public health system and private insurers have their own formularies [23, 25].

National Reimbursement Lists (NRLs) contain medicines selected for coverage (positive list) or specify medicines excluded from reimbursement (negative list). All countries in the WHO European region have at least one reimbursement list, usually in the form of a positive list [9]. The terms “National essential medicines list” and “national reimbursement list” are sometimes used interchangeably but they are not the same unless the NRL is rooted in the EM concept (see Table 1). When NRLs are grounded in the EM concept they have been shown to be an effective tool to ensure the appropriate prescribing of medicines [26, 27]. For example, the well documented Wise List for common diseases in Stockholm County Council, Sweden, has just 200 medicines and has been shown to improve physician adherence and familiarity with medicines and reduce costs [27].

**Table 1:** Priority-setting and implementation tools: definitions, criteria for decision-making and purpose

Tools	Definition	Criteria	Purpose
Priority-setting tools			
WHO Model list of essential medicines (EML)	The Model EML is a select list of medicines developed by the WHO. Essential medicines are those that satisfy the priority healthcare needs of the population [8]	Safety, efficacy, quality, affordability, population need and evidence of market registration	Guides several steps, from production, through procurement to prescribing in primary, secondary and tertiary care settings [5]. It is also used for medicine procurement by UN agencies and international non-governmental organisations (NGOs) [8]
National/Regional/Local EML	National EMLs contain medicines that the country has deemed essential and should be accessible to all people. They may be based on the WHO Model EML. Some countries also have provincial, state or local lists [5]	Safety, efficacy, quality, affordability (cost and budget impact) and population need	Guides several steps, from production, through procurement to prescribing in primary, secondary and tertiary care settings [5]
Health technology assessment (HTA)	A systematic approach to evaluate the properties, effects, and impacts of health technologies; medical devices, medicines, vaccines, procedures, health services, and public health interventions [15, 17, 29]	Budget impact, clinical effectiveness, disease burden, ethical or legal implications, timeliness of review, health economics data and cost-effectiveness [30]	In most national settings, HTA appraisals inform pricing and reimbursement decisions [31]. New technologies, which include medicines, are often compared to the next available alternative
Implementation tools			
Formularies	These are lists of medicines, often organised by drug class or groups of diseases. They contain details of medicines with reference to their uses, cautions, contraindications, side-effects, doses, and costs. They may contain guidance on prescribing, monitoring, dispensing, and administering medicine [5]	Registration status, cost, budget impact and clinical effectiveness	In some settings, a formulary is simply a list of all registered medicines. In others, it aims to guide prescribing behaviour to facilitate effective, efficient, and cost-conscious prescribing. Private insurers may have their own formularies [5]
National reimbursement list (NRL)	A reimbursement list is an instrument used by countries to manage their benefits packages or dictate which medicines will be reimbursed by the government or provided for free [8]	Safety, efficacy, quality, affordability (cost and budget impact) and population need	Provides a list of drugs that are reimbursed by the government and are free to patients. A list may specify medicines selected for coverage (positive list) or those excluded from reimbursement (negative list) [8]
Standard treatment guidelines (STGs)	These are a list of recommendations for medical professionals, normally organised by disease group that specifies which medicines should be used to treat specific illnesses. Countries may develop and use national, regional, or local guidelines [32]	Clinical effectiveness and population need at the relevant level of use	Aims to promote appropriate prescribing behaviour [5]

Terminologies are not standardised and vary by country and systems

### Standard treatment guidelines (STGs)

STGs may be developed in conjunction with the selection process, e.g. in Tanzania, STGs and the national EML are in a combined document [28], or HTA (e.g. by NICE in the UK) to enable rational medicine prescribing. Adherence to STGs should influence appropriate prescribing, consumption, and availability of medicines [5]. The WHO has integrated STGs into the EM concept to inform selection, procurement and prescribing of essential medicines [5]_._ This greatly benefits LMICs with limited capacity for STG development. Treatment guidelines can be developed for use of medicines at different levels of the health system (see Table 1).

#### Deselection and disinvestment

Deselection goes hand in hand with selection strategies undertaken considering new evidence and to prioritise medicines for health budgets. The EM concept has an explicit evidence-based procedure for selection and deselection based on an ideal number of drugs and dosage forms. Applications for addition and deletion of medicines are made to the WHO Essential Medicines Committee and the list is updated every two years. Between the first revision of the EML in 1979 and the 20th edition in 2017, one medicine was deleted for every 2.6 medicines added [33].

While the HTA process can involve disinvestment of medicines that had been previously incorporated into the health system, this is done at country level and without a model list to guide it. Incorporation tends to be a cumulative process. Since additions are not set on population health priorities, but often on needs of individuals or patient groups, and depending exclusively on the existence of evidence of admissible quality, it requires health systems to accommodate a larger set of medicines and much more detailed indications, and at greater cost. The slim evidence on country experience of HTA disinvestment shows limited impact due to strong opposition from the industry (France), limited use (Brazil), or discontinuation of the disinvestment programme (UK) [34–40].

France reviewed all 4490 listed drugs between 2000 and 2004 which were on the market at that time [34, 35] eliminating those with ‘insufficient medical value’; drugs considered dangerous; and drugs no longer considered effective following HTA [35]. Following pharmaceutical industry protests, of the 835 drugs de-listed due to ‘insufficient value’, 763 ‘were re-evaluated between 2003 and 2006, and 238 re-listed [34]. Brazil has HTA guidelines for de-listing medicines or restricting use if they are found to be ineffective or cost-ineffective [36]. In the six years since the guidelines were introduced [37] 220 technologies have been incorporated and 41 have been deselected [41]. In the UK, NICE introduced an active disinvestment pilot programme using HTA in 2005 [38]. It stopped the programme after one year in 2006, concluding that few candidates for de-listing were identified during this process, and that sufficient disinvestment would take place through updating clinical guidelines [38]. There are no published data on how many medicines went through the disinvestment programme or how effective the existing process is. In contrast NICE recommended 32 technologies to be incorporated for use in the period 2006–2007 [39].

## Effectiveness of the two paradigmatic approaches: selection versus incorporation

Medicine prioritisation is transitioning in several countries from traditional selection of essential medicines to incorporation of individual medicines into health systems using HTA [40]. HTA is expanding globally, with India, China and South Africa committing to increasing HTA capacity [42, 43] and Brazil is moving away from the EM concept, towards solely HTA [40]. The EU is moving towards supranational HTA to avoid duplication within the EU [44].

However, there is no research comparing or evaluating the impact of these shifts to HTA on medicines availability, affordability, budgetary impact, and technical capacity. The EM concept, list and STGs are developed by an international team and the Model list is widely adopted because many countries do not have the resources or regulatory capacity to scrutinise the evidence in detail at country level. There is no such process for HTAs where the process and standards differ from country to country. There is also an absence of research into the technical capacity for and effectiveness of HTA as a priority-setting tool or as a cost-containment strategy on its own or as an adjunct to the EM concept.

WHO and Health Action International (HAI) surveys of availability and affordability of medicines show that EMs are more available than those not considered essential in both public and private sectors; more so in low- and lower middle-income countries than upper middle-income countries [38]. However the availability of EMs remains suboptimal globally [7, 45, 46]. Although the EM concept is theoretically focused on cost-containment and affordability resulting from limited lists, the budgetary impact on health systems in countries which use EMLs is unknown.

Most research has focused on auditing the implementation of HTA tools rather than on evaluating and comparing its effectiveness for prioritising medicines, containing costs, ensuring access and appropriate medicine use to meet population needs and with insufficient attention paid to the EM concept. Such research is urgently needed to ensure the EM concept is not lost.

## Data Availability

Not applicable.
